# Avoiding skull radiographs in infants with suspected inflicted injury who also undergo head CT: “a no-brainer?”

**DOI:** 10.1007/s00330-019-06579-w

**Published:** 2019-12-03

**Authors:** Andrew Martin, Michael Paddock, Christopher S. Johns, Jessica Smith, Ashok Raghavan, Daniel J. A. Connolly, Amaka C. Offiah

**Affiliations:** 1 Department of Neuroradiology, Royal Hallamshire Hospital, Glossop Road, Sheffield, S10 2JF UK; 2 Department of Radiology, Barnsley Hospital NHS Foundation Trust, Gawber Road, Barnsley, S75 2EP UK; 3 Academic Unit of Child Health, University of Sheffield, Damer Street Building, Sheffield Children’s NHS Foundation Trust, Western Bank, Sheffield, S10 2TH UK; 4 Department of Radiology, Royal Hallamshire Hospital, Glossop Road, Sheffield, S10 2JF UK; 5 The Medical School, The University of Sheffield, Beech Hill Road, Sheffield, S10 2RX UK; 6 Department of Radiology, Sheffield Children’s NHS Foundation Trust, Western Bank, Sheffield, S10 2TH UK

**Keywords:** Child abuse, Abusive head trauma, Physical abuse, Skull fracture, Tomography, spiral computed

## Abstract

**Objectives:**

To assess whether head CT with 3D reconstruction can replace skull radiographs (SXR) in the imaging investigation of suspected physical abuse (SPA)/abusive head trauma (AHT).

**Methods:**

PACS was interrogated for antemortem skeletal surveys performed for SPA, patients younger than 2 years, SXR and CT performed within 4 days of each other. Paired SXR and CT were independently reviewed. One reviewer analysed CT without and (3 months later) with 3D reconstructions. SXR and CT expert consensus review formed the gold standard. Observer reliability was calculated.

**Results:**

A total of 104 SXR/CT examination pairs were identified, mean age 6.75 months (range 4 days to 2 years); 21 (20%) had skull fractures; two fractures on CT were missed on SXR. There were no fractures on SXR that were not seen on CT. For SXR and CT, respectively: PPV reviewer 1, 95% confidence interval (CI) 48–82% and 85–100%; reviewer 2, 67–98% and 82–100%; and NPV reviewer 1, 95%, CI 88–98% and 96–100%; reviewer 2, 88–97% and 88–98%. Inter- and intra-observer reliability were respectively the following: SXR, excellent (kappa = 0.831) and good (kappa = 0.694); CT, excellent (kappa = 0.831) and perfect (kappa = 1). All results were statistically significant (*p* < 0.001).

**Conclusions:**

CT has greater diagnostic accuracy than SXR in detecting skull fractures which is increased on concurrent review of 3D reconstructions and should be performed in every case of SPA/AHT. SXR does not add further diagnostic information and can be omitted from the skeletal survey when CT with 3D reconstruction is going to be, or has been, performed.

**Key Points:**

*• Head CT with 3D reconstruction is more sensitive and specific for the diagnosis of skull fractures.*

*• Skull radiographs can be safely omitted from the initial skeletal survey performed for suspected physical abuse when head CT with 3D reconstruction is going to be, or has been, performed*.

## Introduction

Abusive head trauma (AHT) is defined as an injury to the skull or intracranial contents from a blunt impact or violent shaking in those children aged under 2 years of age [1]: injuries due to accidental trauma are not included. AHT is a significant finding that has an approximate incidence of up to nearly 40 cases per 100,000 children [2, 3] and can have serious consequences: AHT is the most common cause of death in inflicted injury, comprising 80% of deaths from all head trauma in young children [2]. There is a poor prognosis in survivors with abnormal follow-up in 68% of children: the outcome is correlated with the severity of the injury and 40% have severe neurological deficits [4]. The diagnosis is often missed initially as the presentation of signs and symptoms of an underlying head injury may be delayed [2].

In 2017, the Royal College of Radiologists (RCR) and the Society and College of Radiographers (SCoR) published revised guidelines, endorsed by the Royal College of Paediatrics and Child Health (RCPCH), ‘The radiological investigation of suspected physical abuse in children’ [5]. The European Society of Paediatric Radiology (ESPR) has endorsed the previous [6], current [5], and all future versions of this document as the European standard [7] in the investigation of suspected physical abuse (SPA) in infants and young children. The recently updated guidelines state that all children under the age of 1 year should have multi-slice computed tomography (CT) imaging of the head, in addition to anteroposterior (AP) and lateral skull radiographs (SXR) performed as part of the initial skeletal survey in the investigation of SPA. In children over 1 year of age, a head CT examination is only recommended if there are clinical features suggestive of neurological injury (external evidence of head trauma, abnormal neurological signs and symptoms, or haemorrhagic retinopathy). The guidelines also state that three-dimensional (3D) surface reconstruction of head CT should be performed routinely to better assess for skull fractures and associated soft tissue scalp injury given that head CT with 3D reconstruction is superior to both head CT without 3D reconstruction and SXR [8, 9].

Recent literature has stated that SXR ‘adds little diagnostic value’ to the diagnosis of skull fracture in suspected AHT [10]. Given that head CT has a high sensitivity and specificity for identifying skull fractures, is there still a need to perform SXR if head CT with 3D reconstructions is planned or has been performed? Can we omit SXR from the initial skeletal survey in suspected AHT if head CT with 3D reconstructions is better able to identify skull fractures? We sought to answer these questions by assessing the diagnostic accuracy of head CT and SXR to add to the current evidence pool in the imaging diagnosis of skull fractures in AHT and to inform future iterations of the guidance.

## Materials and methods

### Patients

The picture archiving and communications system (PACS) was interrogated between October 2011 and October 2014 for skeletal surveys which were included if they met the following criteria: antemortem initial skeletal survey performed for non-accidental injury (NAI) (suspected physical abuse, SPA); all children aged less than 1 year of age, and any child less than 2 years of age with neurological symptoms at the time of the initial skeletal survey; SXR and head CT ‘pairs’ performed within 4 days of each other; and imaging performed at our institution (tertiary paediatric neurosciences centre). No follow-up imaging was included. Ethical approval was not required for this retrospective study of anonymised images; however, this study was registered with our local Research and Development and Audit office following which service evaluation approval was granted.

### Image acquisition

The SXR (70 kVp, 2 mAs, 0.008–0.01 mSv depending on patient age) consisted of AP and lateral projections according to our local protocol and the national guidelines [6] at the time of the study. Towne projections were not included, if performed. The head CT examinations consisted of a low-dose non-contrast paediatric head CT performed on a 64-slice GE LightSpeed CT scanner (100–120 kVp, 120–160 mAs, 0.9–3.4 mSv depending on patient age and size, slice thickness range 0.625–2 mm), scanned from the skull vertex to base. Gantry angle was positioned to limit radiation dose to the orbits. Images were anonymised and stored in a training file on the PACS system.

### Image interpretation

The head CT examinations and each set of AP and lateral SXR were reported independently by 2 consultant radiologists (reviewers 1 and 2) with 15 and 16 years’ experience in paediatric neuroradiology. Both were blinded to the clinical details and the originally verified radiological reports. Both skull projections could be viewed at the same time, reflecting clinical practice. The SXR and head CT examinations were given different anonymisation codes and randomised so that the observers could not link the SXR with the corresponding head CT examination. Twenty-two cases were randomly selected and duplicated within the cohort to which the reviewers were blinded, to allow for analysis of intra-observer reliability. Reviewer 1 initially reported all the head CT examinations without 3D reconstructions and then reported all the SXR on a separate occasion. Three months later, this reviewer reported all the head CT examinations for a second time with 3D reconstructions to calculate the diagnostic accuracy of head CT with and without 3D reconstructions. Reviewer 2 reported all the head CT examinations with 3D reconstructions and then reported all the SXR on a separate occasion. Comparison of all SXR and CT with 3D reconstructions for both reviewer 1 and reviewer 2 allowed inter-observer reliability to be assessed.

Separate unique online questionnaires were used by each reviewer which asked them to record if a fracture was present and its location. They were also asked to document if there was any soft tissue injury or sutural diastasis and, in the case of the head CT examinations, if there was any acute intracranial injury. Any disagreements on both head CT and SXR were reviewed at a later time by both reviewers and a consensus opinion was obtained: they were blinded to their original report to allow for unity agreement.

### Statistical analysis

The reference standard was the outcome of the consensus review of SXR and head CT (including 3D reconstructions); patients were considered positive for fracture if the consensus review identified a fracture on one or both modalities. The diagnostic accuracy of each modality was assessed using 2 × 2 contingency tables with calculation of sensitivity, specificity, and positive (PPV) and negative (NPV) predictive values. Inter- and intra-observer reliability was calculated using Cohen’s kappa, with < 0.40 considered poor, 0.40–0.75 considered fair, and ≥ 0.75 considered excellent agreement. A *p* value < 0.05 was considered statistically significant. The data was analysed using the SPSS version 24.

## Results

In total, 104 eligible initial skeletal surveys and head CT examination pairs performed for SPA within 0 to 4 days (mean 0.6 days) of each other were identified. The age range of the 104 children was 4 days to 24 months (mean 6.75 months standard deviation 5.7 months). There were 21 patients with skull fractures (20%), as demonstrated in Table 1.

**Table 1:** Summary of the 21 fracture positive cases

Case	Present on SXR	Present on CT	Side	Type	Sutural diastasis	Intracranial haemorrhage/injury
1	Yes	Yes	Right	Linear	No	No
2	Yes	Yes	Left	Linear	Yes	No
3	Yes	Yes	Right	Linear	No	No
4	Yes	Yes	Left	Linear	No	No
5	Yes	Yes	Right	Linear	No	No
6	Yes	Yes	Right	Linear	No	No
7	Yes	Yes	Right	Linear	No	No
8	Yes	Yes	Right	Depressed	No	SDH
9	Yes	Yes	Right	Linear	Yes	EDH
10	Yes	Yes	Right	Linear	No	No
11	Yes	Yes	Right	Linear	No	SDH
12	Yes	Yes	Right	Linear	Yes	SAH, parietal contusion
13	Yes	Yes	Right	Linear	No	No
14	Yes	Yes	Left	Linear	No	No
15	Yes	Yes	Right	Linear	No	No
16	Yes	Yes	Left	Linear	Yes	SDH
17	Yes	Yes	Left	Linear	No	EDH
18	Yes	Yes	Left	Linear	No	EDH
19	No	Yes	Left	Linear	No	No
20	No	Yes	Right	Linear	No	No
21	Yes	Yes	Left	Linear	No	No

A skull fracture was reported on either radiographs or head CT by consensus in these 21 cases. All 21 fractures were of the parietal bone. Soft tissue (scalp) swelling was present in all cases on radiographs and/or CT

*SXR*, skull radiograph(s); *EDH*, extradural haematoma; *SDH*, subdural haematoma; *SAH*, subarachnoid haemorrhage

### Diagnostic accuracy

Consensus review of the SXR did not identify any skull fractures that were not also seen on consensus review of the head CT examinations. Consensus review of head CT identified two fractures not seen on consensus review of SXR (Table 1). The diagnostic accuracy was higher for both reviewers for head CT reporting compared with that for SXR, with higher sensitivity and specificity. Diagnostic accuracy for the reporting of skull radiographs and head CT, with and without 3D reconstructions, for both reviewers is displayed in Table 2, with separate diagnostic accuracy for reviewer 1 in Table 3.

**Table 2:** Diagnostic accuracy for the reporting of skull radiographs and head CT, with and without 3D reconstructions, for both reviewers

	Reviewer 1			Reviewer 2	
	Radiograph	CT	CT with 3D	Radiograph	CT
Sensitivity	81% (95% CI 60–92%)	81% (95% CI 60–92%)	100% (95% CI 85–100%)	77% (95% CI 55–89%)	81% (95% CI 60–92%)
Specificity	90% (95% CI 82–95%)	99% (95% CI 93–100%)	100% (95% CI 96–100%)	98% (95% CI 92–100%)	100% (95% CI 96–100%)
PPV	68% (95% CI 48–82%)	94% (95% CI 74–100%)	100% (95% CI 85–100%)	89% (95% CI 67–98%)	100% (95% CI 89–98%)
NPV	95% (95% CI 88–98%)	95% (95% CI 89–98%)	100% (95% CI 96–100%)	94% (95% CI 88–97%)	95% (95% CI 89–98%)

*p* < 0.001 for all results; *CI*, confidence interval

**Table 3:** Diagnostic accuracy for the reporting of head CT, with and without 3D reconstructions, for reviewer 1

	Reviewer 1 without 3D reconstructions	Reviewer 1 with 3D reconstructions
Sensitivity	81% (95% CI 60–92%; *p* < 0.001)	100% (95% CI 85–100%)
Specificity	99% (95% CI 93–100%; *p* < 0.001)	100% (95% CI 96–100%; *p* < 0.001)
PPV	94% (95% CI 74–100%; *p* < 0.001)	100% (95% CI 85–100%; *p* < 0.001)
NPV	95% (95% CI 89–98%; *p* < 0.001)	100% (95% CI 96–100%; *p* < 0.001)

*p* < 0.001 for all results; *CI*, confidence interval

**Fig. 1:**
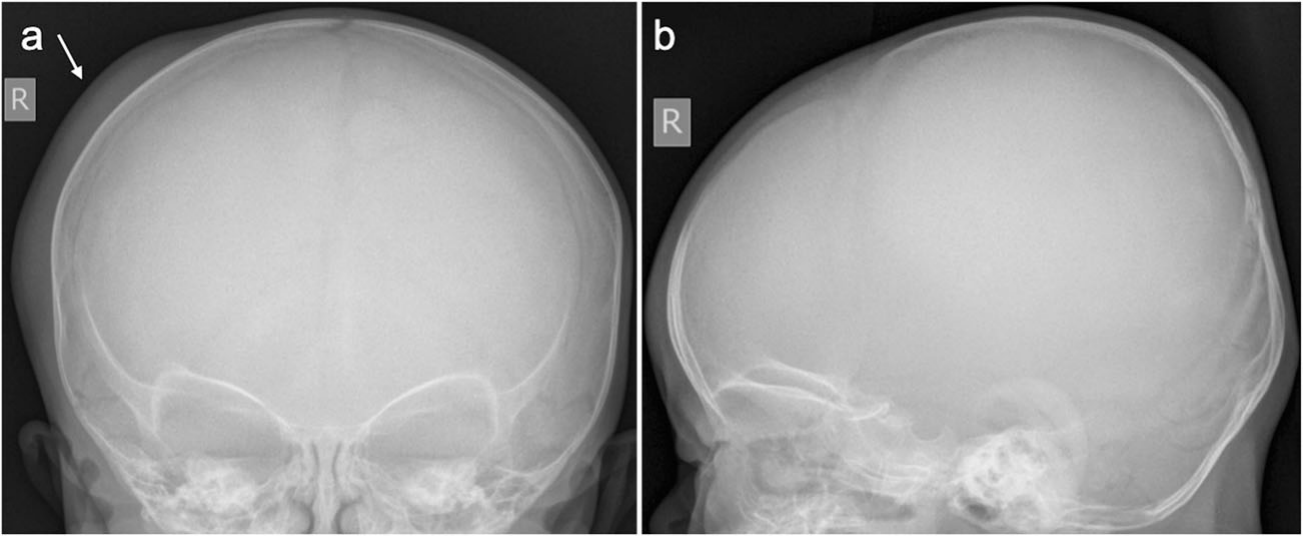
AP (**a**) and lateral (**b**) skull radiographs of a 16-week-old infant (case 20) following a reported fall. There is soft tissue swelling over the right side of the head (arrow), but no fracture is identified

**Fig. 2:**
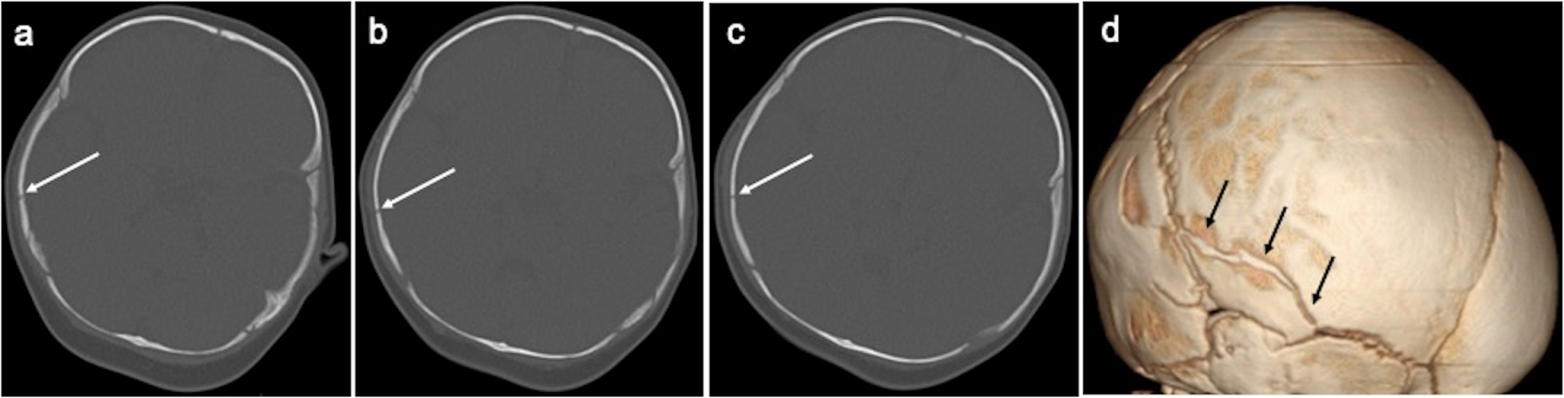
**a**–**c** Selected axial slices (inferior to superior) on bone windows from the head CT in the same infant as Fig. 1 which demonstrate a fracture of the right parietal bone (white arrows) with overlying soft tissue swelling. The corresponding right lateral view of the 3D reconstruction (**d**) demonstrates the fracture in the right parietal bone (black arrows) which extends to the right squamoparietal suture

**Fig. 3:**
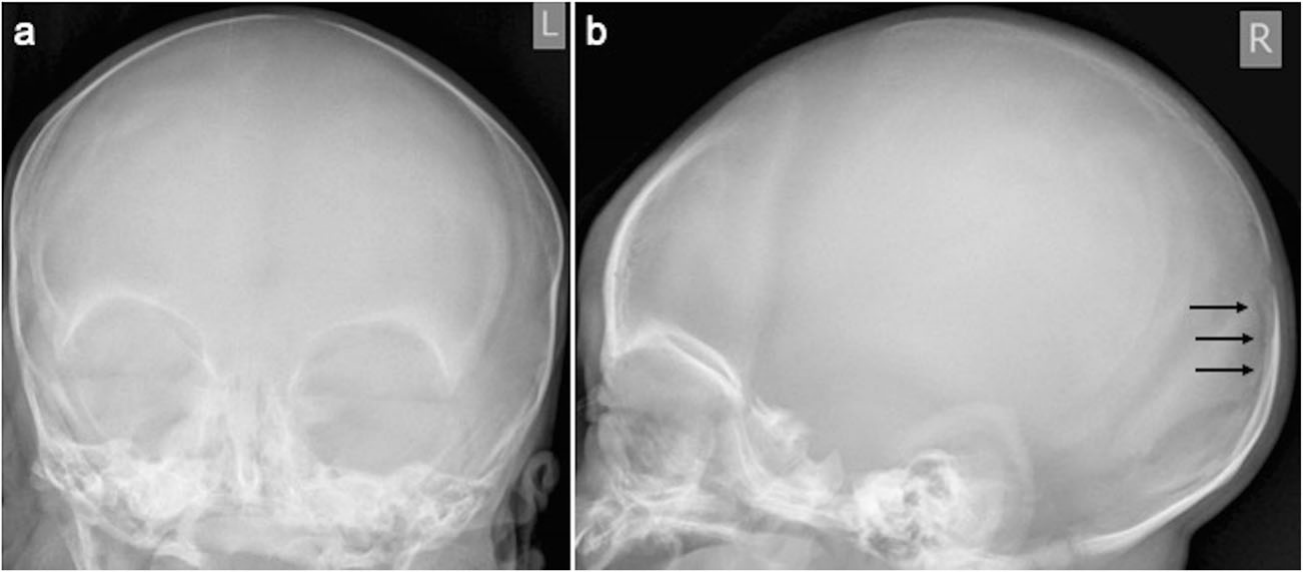
False-positive fracture on radiography: AP (**a**) and lateral skull (**b**) radiographs of a 24-day-old infant. There is a linear lucency in the occipital bone (black arrows) which was reported as a fracture by one of the reviewers

**Fig. 4:**
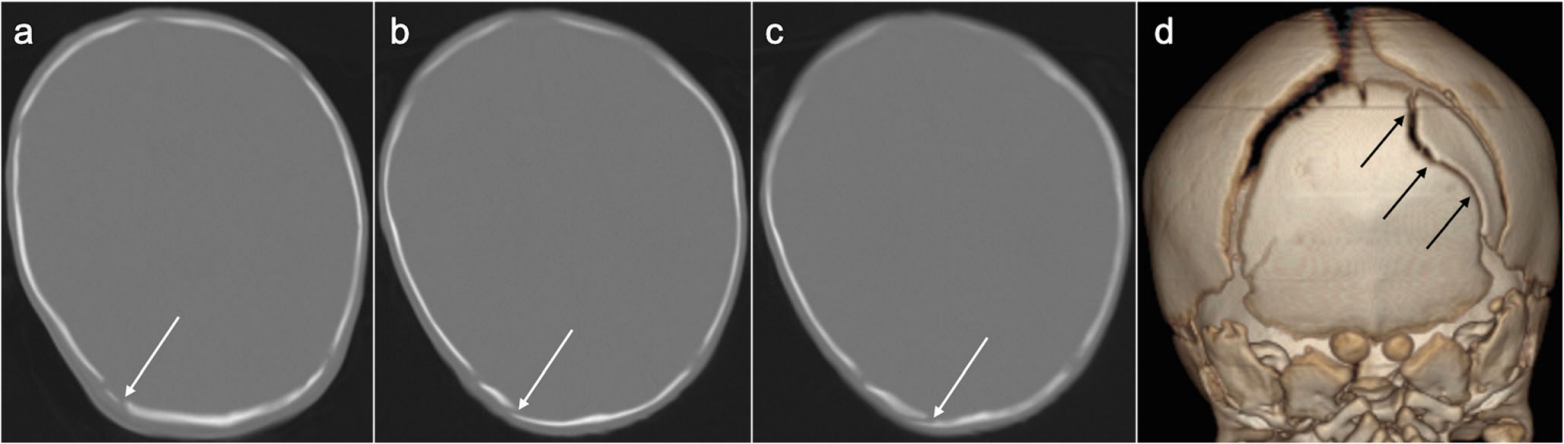
**a**–**c** Selected axial slices (inferior to superior) on bone windows from the head CT examination in the same infant as Fig. 3 which demonstrate a cortical irregularity involving the right occipital bone (white arrows), suggestive of a fracture. However, there is no associated soft tissue swelling and on the 3D reconstruction (**d**); the cortical irregularity is curvilinear in appearance rather than linear, the latter being more typical of a fracture. The location, adjacent to the lambdoid suture, its appearance, and the absence of soft tissue swelling suggest this is more in keeping with an accessory lambdoid suture (black arrows)

Figures 1 and 2 illustrate a skull fracture that was not demonstrated on SXR (Fig. 1), but which was evident on the corresponding head CT and 3D reconstruction (Fig. 2), as determined by reviewer consensus. On independent review, eight fractures were identified on SXR that were not confirmed by the reference standard (i.e. false positives), of which two were accessory lambdoid sutures (Figs. 3 and 4); one was an accessory coronal suture; three were accessory sagittal sutures; one was a vascular channel in the parietal bone; and in the final case, it was unclear as to what reviewer 1 had interpreted as a fracture.

There was excellent inter-observer (kappa = 0.831, 95%CI 0.668–1.000, *p* < 0.001) and fair intra-observer agreement (kappa = 0.694, 95%CI 0.299–1.000, *p* < 0.001; reviewer 2: 0.70 95%CI 0.32–1.00, *p* < 0.001) for the reporting of fractures on skull radiography. There was excellent inter-observer (kappa = 0.831, 95%CI 0.668–1.000, *p* < 0.001) and perfect intra-observer agreement (kappa = 1.000, 95%CI 1.000–1.000, *p* < 0.001) for both reviewers for the reporting of skull fractures on head CT.

## Discussion

Our study showed that head CT has a high sensitivity and specificity for the diagnosis of skull fracture. 3D reconstruction of the skull and multiplanar reformatting (axial, coronal, and sagittal views), when viewed on bone window, provide improved visualisation of fractures that may be missed in the axial plane of the CT slice. Head CT also allows assessment of soft tissue swelling, which may be overlooked on SXR. Previous studies have compared radiographs and head CT for the diagnosis of skull fractures but have their limitations.

The mean age of children in the study by Orman et al [11] was 7.8 years, outside the normal range for SPA. The age range of the 42 post-mortem patients recruited by Chawla et al [12] was not stated. Sharp et al [13] documented contemporaneous radiology reports rather than reviewing SXR and head CT and seeking a consensus opinion. Furthermore, the rank of reporter was not specified (i.e., consultant or trainee/resident) and intra- and inter-observer reliability were not reported. In this study, we sought to address these limitations.

The mean age of the children we recruited (6.75 months) reflects the age for AHT/SPA. Our study design allowed inter- and intra-observer reliability to be determined and we compared diagnostic accuracy with and without 3D reconstruction from head CT. We have demonstrated that head CT has greater diagnostic accuracy and is more reliable than SXR in the assessment of skull fractures in infants and young children. Furthermore, we have demonstrated that the diagnostic accuracy of head CT is improved when there is concurrent analysis of 3D reconstructions. Our data shows that head CT is at least as sensitive and as specific as SXR when 3D reconstructions are not used (CT vs SXR = sensitivity, 81% vs 77–81%; specificity, 99% vs 90–98%) but more sensitive and specific when 3D reconstructions are used (CT vs SXR = sensitivity, 81–100% vs 77–81%; specificity, 100% vs 90–95%). Our data also demonstrated that intra- and inter-observer agreement is improved when reporting skull fractures on head CT when compared with those on SXR with perfect and excellent agreement, respectively. This highlights the ambiguity of diagnosing fractures on SXR when compared with the certainty that head CT provides when reporting skull fractures. Chawala et al [12] assessed sensitivity and specificity of head CT by comparing antemortem head CT with post-mortem autopsy findings and demonstrated a sensitivity of 85% and a specificity of 100% in the diagnosis of skull fractures. Sharp et al [13] assessed whether the use of SXR was still justified as part of the imaging investigation for suspected AHT/SPA (i.e. the initial skeletal survey) when a head CT was being performed. Of their 94 patients, they found that SXR demonstrated no additional findings, but that additional findings were demonstrated in 2 cases on head CT. They concluded that SXR could be excluded from the initial skeletal survey if volumetric head CT examinations were also being performed.

Our results support this view and are also in line with those from a study performed in 2017 by Culotta et al [10], who found that head CT examination with 3D reconstruction was equivalent to SXR in identifying skull fractures in suspected AHT. Whilst the Culotta et al [10] study had a larger cohort of 177 patients, the mean and median age of children was 5 months, whereas our study included children up to the age of 2 years which better reflects the age range of those children investigated for suspected AHT/SPA in clinical practice, and as stated in the latest published national and European guidelines [5]. Moreover, they compared the sensitivity of head CT with 3D reconstructions with that of SXR, whereas we evaluated the diagnostic accuracy of head CT, with and without 3D reconstructions, against the reference standard (consensus review of SXR and head CT where patients were considered positive for fracture where the consensus review identified a fracture on one or both modalities). Orman et al [11] found that the use of 3D reconstruction increased both sensitivity and specificity in the diagnosis of linear skull fractures in children when compared with conventional axial CT (83.9% vs 78.2% and 97.1% vs 92.8%, respectively). Significant advantages of using 3D reconstruction include no increased radiation burden, no additional scan time, and availability at no extra cost. Furthermore, CT has established itself as a problem-solving tool in differentiating skull fractures from common anatomical variants (e.g. accessory sutures) with a greater sensitivity than radiography [14], as also demonstrated in our study.

There is ongoing research on the use of novel techniques to better evaluate skull fractures on magnetic resonance imaging (MRI). Dremmen et al [15] compared CT and a black bone MRI sequence in 28 children following head trauma. Black bone sequences use an ultrashort TE and TR to minimise the signal returned from soft tissues which enhance the bone-soft tissue interface. They found that MR imaging with the black bone sequence had a lower sensitivity (66.7%) and specificity (87.5%) than CT. They concluded that its use is a promising alternative but the detection of linear fractures, particularly in aerated bone, remains limited. A subsequent study by Kralik et al [16] evaluated 34 patients with suspected AHT using CT with 3D reconstruction and a black bone MRI sequence with multiplanar reconstructions and 3D volumetric images. They found that black bone imaging had a sensitivity of 83% with 100% specificity and detected 95% of the skull fractures that were visualised on CT. Whilst these results are encouraging as MRI obviates the need for exposure to ionising radiation, the cohorts investigated are small, the technique may not be available in all centres at all times (particularly in the general hospital setting where the majority of children are presented), and black bone MRI sequences still miss some fractures.

Due to insufficient evidence upon which to base a change in practice at the time, the recently updated guidelines for the investigation of SPA (like the previous edition) recommend performing AP and lateral SXR as part of the initial skeletal survey, even if head CT is performed. Guideline 35 states that, ‘3D surface reconstructed images employing bone and soft tissue windows should be undertaken for better appreciation of skull fractures and associated scalp soft tissue injuries. This does not replace the need for AP and lateral skull radiographs, which provide complementary information’ [5]. Previous thinking has been that although SXR provide no information with regard to intracranial injury, they may help with the identification of skull fractures, particularly linear fractures occurring in the plane of the head CT slice which may not be readily identifiable. In our study, there were no fractures on SXR that were not also visualised on head CT. Thus, SXR confer no diagnostic benefit when head CT with 3D reconstructions are available: removing AP and lateral SXR from the initial skeletal survey when a head CT is going to be or has been performed would reduce radiation dose and distress to the patient, in addition to saving time. There is also the additional benefit of being able to identify intracranial pathology which would not be evident on SXR and may be clinically silent until the patient deteriorates, such as intracranial haemorrhage. In the 21 cases where a fracture was identified, 7 also had either a subdural or extradural haematoma (Table 1).

In the imaging investigation of SPA, it is recommended that all children below 1 year of age have head CT, whilst a head CT examination is only recommended if there are clinical features of neurological injury (external evidence of head trauma, abnormal neurological signs and symptoms, or haemorrhagic retinopathy) in children aged between 1 and 2 years. Currently, both groups of children will have AP and lateral SXR performed as part of their initial skeletal survey. Given the results of our study, we recommend that head CT should replace SXR in the imaging investigation of SPA in children under the age of 1 year and those over 1 year of age who present with neurological injury. Until further evidence is available, for those children over the age of 1 year without abnormal neurological signs and symptoms, we recommend that the national guidance should be followed and that SXR should continue to be employed as part of the initial skeletal survey unless head CT has been, or is going to be, performed.

### Limitations

This was a retrospective observational cohort study. As such, we were dependent on the dose, imaging parameters, and quality of imaging at the time of acquisition.

We are aware that infants and young children with suspected AHT may not be presented to a tertiary paediatric neurosciences centre, such as our institution. However, the use of CT in the imaging investigation of acute head injury in children is well established, as is the wide availability of the 3D reconstruction software which may facilitate interpretation by non-radiologists (i.e. emergency medicine physicians) when radiologists are unavailable. The two reviewers in this study were consultant paediatric neuroradiologists with extensive clinical experience; however, this may not reflect real-life clinical practice where general or non-specialist radiologists may report head CT imaging, particularly out-of-hours, where acute head CT imaging may be more commonly reported by trainee/resident radiologists. We could have investigated this by including a third, less-experienced observer. Although the number of reviewers may be seen as a limitation, the results are compelling.

Whilst the number of patients in our study is not as large as that in Culotta et al [10], the age of the patients in our cohort is more reflective of those that would be investigated in clinical practice, in addition to addressing several of the limitations of the other published studies, as discussed above.

## Conclusion

In this relatively large study of infants and children below 2 years of age, the first to contextualise the diagnostic accuracy of head CT and SXR since the publication of the revised RCR guidelines endorsed by ESPR for use throughout Europe, we have demonstrated that (1) diagnostic accuracy is greater for head CT than for SXR in the detection of skull fractures; (2) the routine use of SXR in the imaging investigation of SPA does not add further diagnostic information; and (3) concurrent review of 3D reconstructions increases the diagnostic accuracy of CT and should be performed in every case of suspected AHT/SPA. We conclude that head CT can replace SXR in the investigation imaging of SPA in children under 1 year of age and in those over 1 year of age who present with neurological injury.

## Abbreviations

3D: Three-dimensional
AHT: Abusive head trauma
CI: Confidence interval
CT: Computed tomography
ESPR: European Society of Paediatric Radiology
MRI: Magnetic resonance imaging
NAI: Non-accidental injury
NPV: Negative predictive value
PACS: Picture archiving and communications system
PPV: Positive predictive value
RCPCH: Royal College of Paediatrics and Child Health
RCR: Royal College of Radiologists
SCoR: Society and College of Radiographers
SPA: Suspected physical abuse
SXR: Skull radiograph(s)
